# Modelling and interpreting fish bioenergetics: a role for behaviour, life-history traits and survival trade-offs

**DOI:** 10.1111/jfb.12834

**Published:** 2016-01-14

**Authors:** C Jørgensen, K Enberg, M Mangel

**Affiliations:** * Uni Research and Hjort Centre for Marine Ecosystem DynamicsP. O. Box 7810, 5020, Bergen, Norway; ‡ Institute of Marine Research and Hjort Centre for Marine Ecosystem DynamicsP. O. Box 1870 Nordnes, 5817, Bergen, Norway; § Center for Stock Assessment Research, University of California Santa CruzSanta Cruz, CA, 95064, U.S.A.; ∥ Department of Biology, University of BergenP. O. Box 7803, 5020, Bergen, Norway

**Keywords:** aerobic scope, metabolic costs, mortality, non-consumptive effects, risk-taking, SMR

## Abstract

Bioenergetics is used as the mechanistic foundation of many models of fishes. As the context of a model gradually extends beyond pure bioenergetics to include behaviour, life-history traits and function and performance of the entire organism, so does the need for complementing bioenergetic measurements with trade-offs, particularly those dealing with survival. Such a broadening of focus revitalized and expanded the domain of behavioural ecology in the 1980s. This review makes the case that a similar change of perspective is required for physiology to contribute to the types of predictions society currently demands, *e.g*. regarding climate change and other anthropogenic stressors.

## Introduction

This paper describes how models may put fish bioenergetics in a broader context and thus contribute to making the discipline of physiology more useful for science and society. Models are the key tools for translating animal physiology into predictions that can be taken up or have effect outside the discipline itself (Tomlinson *et al.*, 2014; Mangel, 2015). Models can harness the predictive ability of acquired insights and provide scenarios that are useful for societies that want to know what might happen, with a species or ecosystem, in response to local or global perturbations. Models often need to embed a particular discipline at par with other biological subject areas, however, to arrive at the types of predictions society desires. Through such a process, fish physiology might become part of the information base for policy-making and interplay between physiologists, behavioural ecologists, ecosystem scientists and modellers is often necessary to achieve this.

There are many types of models, each being able to make certain predictions from a set of assumptions. Models therefore vary in how well they can address a given scientific problem. This review attempts to draw parallels between physiological experiments and modelling methods, as a guide to the complex landscape of models and their strengths and weaknesses. It also points to areas where further model development hinges on advances within physiology, with the hope that this can stimulate physiological research.

## Different approaches to measuring fish bioenergetics

### First approach: standard metabolic rates of resting fishes

**Figure 1:**
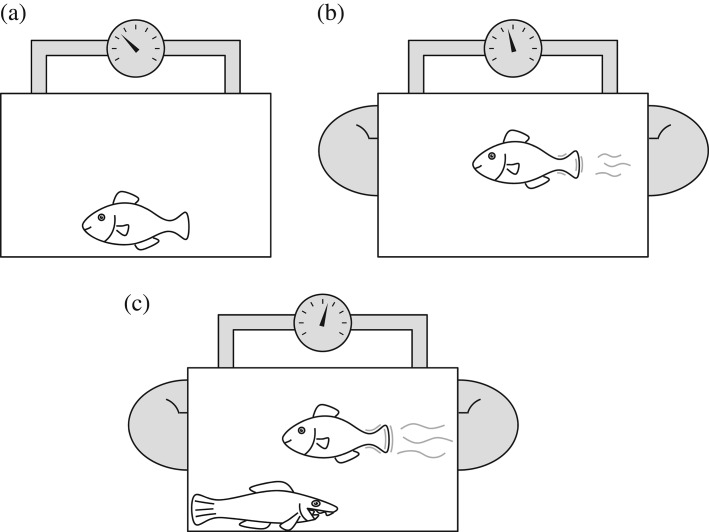
How well do measures in the laboratory approach a real-life setting with predators and trade-offs? (a) Basal or standard metabolic rate quantifies the cost of living while an individual does nothing. This is problematic as it is usually hard work to obtain fitness, where one has to be successful at foraging, growth, survival, mating and reproducing. (b) A more complex picture is painted as costs of various physiological functions can be determined, in particular this has been extensively done for swimming. Locomotion is required for many vital functions in life, and these kinds of estimates are needed to piece together a full-fledged energy budget. (c) By modifying perceived predation risk, trade-offs and non-consumptive effects of predation can be quantified as they affect voluntary rates of, for example, swimming and foraging. Visual contact with a predator or water that smells of predators can induce behavioural changes that can be quantified. This type of laboratory or field-experiment approaches the real-life setting where energetics and survival are joint factors that over time have influenced the adaptation of behaviours and life histories currently observed.

Imagine a physiological experiment quantifying bioenergetic costs of a fish kept in a laboratory. A widely used approach is to measure the minimum cost of living as the standard metabolic rate (SMR; Chabot *et al.*, 2016a). To achieve this, an acclimated, fasting and non-swimming fish is kept in a respirometer to quantify oxygen consumption [Fig. 1(a); Chabot *et al.*, 2016a]. One utility of this measure is that it is comparable across taxonomic groups, between species with different physiologies or ecologies or between different environments. Strong empirical scaling relationships have been revealed with body size (the so-called mouse to elephant curve; Kleiber, 1932; Winberg, 1960; Schmidt-Nielsen, 1984) and temperature (Clarke & Johnston, 1999; Brown *et al.*, 2004). Less is known about whether within-species relationships follow the same scaling as between-species measurements (Schmidt-Nielsen, 1984; Killen *et al.*, 2010). A closer look at the measurements reveals that, beyond the scaling relationships with size and temperature, SMR also depends on taxonomic group (Brown *et al.*, 2004), endothermy *v*. ectothermy (Brown *et al.*, 2004), diet (McNab, 1986, 1988), swimming style (Killen *et al.*, 2010) and a range of other physiological and ecological measures (White & Seymour, 2004). Some of the differences in SMR can thus be ascribed to the ecology or life history of a species, but the question of which processes and costs are included, willingly or not, in the measurement of the trait remains open. The strong signal of food habits such as herbivory, frugivory, insectivory and carnivory (McNab, 1986, 1988), for example, suggests that the digestive tract, and possibly also detoxification organs, incur maintenance costs that vary according to the task they routinely perform (in addition to the elevated metabolic rate in response to a particular meal; Chabot *et al.*, 2016b). And while the cost of the digestive system is a continual and constant investment in most species, there are examples where the digestive tract is atrophied and rebuilt according to need (Dekinga *et al.*, 2001; Secor, 2008). Just as there are species-specific differences in the long-term energetic costs of maintaining the digestive system, there are species-specific differences in other organ systems and for more short-term energetics too. Species vary in their ability and propensity to move, chase, hunt or migrate, and the major part of the associated energetic cost is paid only during the activity itself, to a much lesser extent when at rest. In addition, many other functions are inseparable from SMR, such as detoxification, immuno-competence, sensing and cognition, toxin production or signalling and armoury (Enberg *et al.*, 2012). While SMR is an important baseline that allows interpretation and modelling of a species' bioenergetic budgets, it is conceptually not clear why one would want to compare, across species, a measure that includes costs of some adaptations and life-history traits but not others.

### Second approach: costs of locomotion and other activities

One way to amend this situation is not only to consider SMR but also to quantify costs of several regular activities a fish may undertake to increase its Darwinian fitness, for which expected lifetime reproductive success is often a good proxy (Mangel, 2015). This is illustrated by the swimming respirometer in Fig. 1(b) in which oxygen consumption can be measured at different swimming speeds. Put in the context of optimal cost of transport, cruising speed while foraging, escape responses or sexual display, locomotion and its costs can be directly relevant for many activities that are important for fitness (Domenici & Kapoor, 2010). Indeed, for swimming, several methodologies exist to paint a fairly complete picture of costs and efficiencies during steady state (swimming respirometry; Brett, 1964) and also for more short-term locomotory behaviours (stereo-video, Tang & Boisclair, 1995; high speed video analysis, Domenici & Blake, 1997; digital particle image velocimetry, Drucker & Lauder, 1999). For other traits, the energetic costs are less well described and quantified, *e.g*. swimbladder buoyancy regulation (Strand *et al*., 2005), sexual display, production of sperm or eggs, parental care or being a victim of diseases or parasites. For models to paint a fuller picture of organism design as being adaptive, it would be desirable to know the costs and benefits of many traits. Beyond the technical difficulties of disentangling costs of traits that have both short-term and long-term effects, *e.g*. how to measure separately the investment in maintenance of the digestive tract from maintenance of the immune system, there appears to be a bias in traits that are measured towards those for which comparative data already exist.

### Third approach: quantifying flexible responses and trade-offs

Contemplate then how energetic costs may change as the fish modifies its behaviour in the presence of a predator, as is illustrated by Fig. 1(c). It is likely that even the most sophisticated measures of bioenergetics fall short of prescribing fitness consequences in the wild, and the reason is one that transformed behavioural ecology in the 1980s: slightly simplified, fitness can be defined as the product of the resources that bioenergetics can channel towards reproduction multiplied by the survival probability until that age. In behavioural ecology, the integration of these two dimensions, survival and foraging rate, was labelled as ‘the common currency’ for behavioural decisions (McNamara & Houston, 1986; Houston *et al*., 1988; Mangel & Clark, 1988). Not only did this spawn new methods and abundant theoretical and empirical research (Houston & McNamara, 1999; Clark & Mangel, 2000), but also the central role of survival forced a clearer focus on trade-offs and allowed integration with life-history theory (Roff, 1992; Stearns, 1992). Performing laboratory experiments that reveal these trade-offs is challenging, and perhaps one fruitful way is to extend Krogh's principle (Krogh, 1929) to the ecological dimension. Phrased in modern language, Krogh (1929) stated that, ‘among the diversity of animal species there will be one ideally suited as an experimental model for any biological problem’ (Lindstedt, 2014). Physiologists, particularly eco-physiologists, have successfully adhered to the Krogh principle, but studies of trade-offs linking physiology and ecology are rarer. One inspiring example is the series of studies on Atlantic silversides *Menidia menidia* (L. 1766). Using a natural gradient along the U.S. east coast, fish adapted to high latitudes, where the summer growing season is shorter, have a higher motivation to grow fast, voluntarily ingest larger meals and set aside more of their oxygen budget to digestion and biosynthesis, but as a consequence of the high growth motivation these fish suffer higher predation rates (Conover & Present, 1990; Billerbeck *et al*., 2001; Lankford *et al*., 2001; Arnott *et al*., 2006; Chiba *et al*., 2007). This links physiology, behaviour and life-history traits through trade-offs in an exemplary way. More recently, consistent among-individual variation in SMR and active metabolic rate has been linked to behavioural types, where fishes with high metabolic expenditure typically are bold and grow fast when food is abundant but lose this advantage in times of scarcity, when they may take even more risks and as a consequence have lower survival (Metcalfe, N. B. *et al*., 2016). It should be possible to quantify similar trade-offs in more species by viewing physiology as the set of rules and constraints, behaviour as mediating a flexible strategy and life-history traits as providing the framework of interpretation. This would expose the important constraints under which fish design has evolved.

## Parallels between measured bioenergetics and modelling methods

Many frameworks using bioenergetic modelling are in use today, and with the risk of some simplification these can be grouped according to the experimental protocols in the previous section.

### Metabolic theory of ecology (parallel to the first approach)

The metabolic theory of ecology (MTE; Brown *et al*., 2004) takes as its basis the scaling of basal (or standard) metabolism [*e.g*. as measured in Fig. 1(a)] across all taxonomic groups of life and its variation with size, temperature and other factors. As a null model for ecology this is very useful (Harte, 2004), but problems start to accumulate as the focus extends to traits other than SMR. Huge variation exists among species of the same size, and when only a limited size range is considered the predictive power of body size alone vanishes (Tilman *et al*., 2004). For an empirical data-mining study that looks for broad-scale patterns this does not matter much, but many of the assumptions remain untested (Price *et al*., 2012) and problems begin to accumulate if MTE is used for predictive purposes. For example, in their review of MTE, Brown *et al*. (2004) included body size in the driver's seat not only of large-scale ecological patterns but also of life-history traits specific to a species. They write that life histories can be adaptive because ‘organisms can respond to selection resulting from different environments by changing body size’ (Brown *et al*., 2004). Thus, the MTE framework views metabolic scaling as universal, body size as a master trait and everything else as consequences. Fish physiologists would disagree with such a world-view, having studied fish species of similar size with vastly different solutions for, for example, foraging, locomotion and reproduction. While size is no doubt an important trait, there are many other factors that also have strong influences on an organism's physiology, behaviour, life history and ecology. Furthermore, fish body size is highly plastic and in many species there is considerable growth after maturity, depending upon food resources.

A genealogy of models has been derived from MTE and, for illustration, models aiming to predict individual growth are considered here. Hou *et al*. (2008) and Hou (2014) used the assumptions of MTE to derive growth models that fit observed growth trajectories of a range of mammals astonishingly well. Modelled growth curves lie exactly on top of observed data points, and based on goodness of fit the models make predictions for compensatory growth, cellular damage and senescence. It is, however, problematic that the models omit reproduction altogether, even though reproduction is a major energetic cost for most species with huge tolls on their metabolic budgets. Also, from the perspective of evolution, strategies that do not involve reproduction will never make it to the next generation. Until these models account for reproduction in a way that is consistent with fitness and natural selection, the goodness of fit with data can only be interpreted as there being a huge systematic error somewhere else in the model. It is time to abandon MTE-based models as a basis for making predictions for species or individuals; the scale where MTE has any relevance is vastly bigger. Alternatives are available; for example, the models by Mangel & Munch (2005), Mangel (2008) and Bonsall & Mangel (2009) address many of the same phenomena as Hou *et al*. (2008) and Hou (2014) but do so while including reproduction and being consistent with evolutionary processes and Van Leeuwen *et al*. (2010) provide a dynamic energy budget (DEB) model of physiological mechanisms.

### Swimming respirometry or costs of single functions (parallel to the second approach)

Activity, including locomotor activity, often accounts for a large proportion of a fish's energy expenditure because it is so much more costly than SMR. Just as fish in the second approach [Fig. 1(b)] are performing important activities when the energetic costs are quantified, a range of models have taken a more nuanced view of how an energy budget is a sum over multiple activities and dynamic over time. One important development was the widespread application of the Wisconsin model of fish bioenergetics (Hewett & Johnson, 1987, 1992; Hanson *et al*., 1997), particularly for freshwater species in North America but also for many marine species. There is nothing mysterious about the equations of the model; they just do the bookkeeping of energetic processes from ingestion and digestion through to waste, metabolism and growth, and the software package made the model easy to apply. As the model was fitted to gradually more and more species, missing parameters in one species could be borrowed from a related or ecologically similar species.

In parallel and somewhat later, Kooijman (1993, 2010) developed the DEB approach. While the Wisconsin model had its focus on empirically estimated rates and practical consequences for growth, population dynamics and fisheries, the goal of DEB was to arrive at a fundamental description of bioenergetics, with universal applicability to all organisms. This ambitious aim required a more theoretical and deductive approach, with abstract assumptions about the fundamental organizational properties of all life. Although the route taken was diametrically opposite, the assumed similarity across organisms had the same consequence: what was not known in one species could be assumed from knowledge of other species. More recently, the ‘Add My Pet’ software (www.bio.vu.nl/thb/deb/deblab/add_my_pet/Species.html) goes further by allowing estimation of DEB parameters from empirical data sets while borrowing unknown parameters from related species.

DEB shares many characteristics with MTE; both are tools aiming at describing bioenergetics as a unifying principle applicable across all domains of life. They also differ: while MTE focuses on one point per species and uses broad comparisons, DEB is explicit about how processes change through an individual's lifetime due to ontogeny, growth and reproduction. There are also other physiological models that go far in the direction of biochemical and physiological detail (Bar *et al*., 2007); these often do not fall within a common framework but are designed on a species by species basis and often for more particular or applied purposes.

Another suite of models focuses on the local physiological implications of a given spatio-temporal map of the environment, *e.g*. locomotion and the specific ecology of stream-dwelling salmonids (Railsback *et al*., 2012, 2013). Kraus *et al*. (2015) combined bioenergetic models with observations of environmental temperature and oxygen to backcalculate habitat-specific growth potential for striped bass *Morone saxatilis* (Walbaum 1792), and found that observations of fish in a warmer-than-optimal estuary could be explained by high food abundance. Another example is the role of local temperature and oxygen availability on aerobic scope in the grey mullet *Mugil cephalus* L. 1758 (Cucco *et al*., 2012). The *M*. *cephalus* migrates between the well-aerated sea and semi-enclosed lagoons where temporary hypoxia may render the habitat unfavourable. Coupling environmental characteristics with physiological response functions provides much deeper insight into drivers of movements and life cycles. A challenge is that predation risk is seldom incorporated in these analyses, and needs to be weighed against the energetic profitability of each environment. How, for example, would the *M*. *cephalus* choose between an oxygenated environment that allows high metabolic activity to sustain rapid growth and reproduction but contains numerous predators *v*. a hypoxic habitat that at times puts severe constraints on physiological performance but where predation is lower? In the case of *M*. *cephalus*, there might be additional motivation that attracts them to the lagoon, as they also spawn there. To solve this problem, it is necessary to move beyond considering foraging efficiency in isolation (Emlen, 1966; MacArthur & Pianka, 1966; Charnov, 1976) towards discounting the fitness benefit of any behavioural decision with the risk it entails in terms of decreased survival (Houston *et al*., 1988).

### Trade-offs involving survival (parallel to the third approach)

A common characteristic shared by the modelling approaches above is that they represent the fish as if it were in a predator-free environment, often inspired by a laboratory experiment. In nature, predators not only occasionally lead to death, but also prey species that generally show flexible behaviours (Dill & Fraser, 1984; Chiba *et al*., 2007) or morphology (Brönmark & Miner, 1992) depending on the risk imposed by the predator community.

For example, the presence of predators can make prey choose behaviours or environments that in a predator-free world would be suboptimal (McNamara & Houston, 1987; Creel & Christianson, 2008); this is often referred to as predators having non-consumptive effects (Peacor *et al*., 2013). Behaviour under threat of predation has been addressed conceptually in reviews (Lima & Dill, 1990; Creel & Christianson, 2008) and specifically in experiments (Dill & Fraser, 1984; Chiba *et al*., 2007).

In modelling, fitness consequences of a given behaviour had to be discounted by survival, which introduced the common currency for behaviours (McNamara & Houston, 1986; Houston *et al*., 1988). Models that explicitly account for predation effects can be roughly separated into two groups. First are those that study short-term behaviour (McNamara & Houston, 1987; Clark & Levy, 1988; Rosland & Giske, 1994; Fiksen & Jørgensen, 2011). These are to various degrees driven by physiology and energetics, and focus on the behavioural trade-offs between foraging efficiency and risk exposure. Another group of models use much coarser time-steps and consider effects of mortality on evolution of life-history traits, often motivated by industrial fishing as a new evolutionary driver (Law & Grey, 1989; Hutchings, 2005; Jørgensen & Fiksen, 2006, 2010; Dunlop *et al*., 2009; Enberg *et al*., 2009).

All the models involving effects of mortality and predation draw on biological mechanism, including physiology. Physiology is, however, often used as part of trade-offs where a physiological cost (energetic costs) or benefit (foraging and growth) on one side is traded against risk of predation on the other. Many of these trade-offs were reviewed in light of their consequences for growth (Arendt, 1997; Enberg *et al*., 2012) but the logic can be extended to bioenergetics more generally. Often, the standard approaches for physiological modelling do not extend easily to evolving traits or optimization of behaviours, but models incorporating behavioural trade-offs with predation (Plumb *et al*., 2014) and for life-history evolution (Kooi & van der Meer, 2010) have also been formulated within the DEB framework.

To appreciate the interplay between ecology and physiology, and areas where more research is needed, consider recent models of climate evolution in a boreal fish species, cod *Gadus morhua* L. 1758 (Holt & Jørgensen, 2014, 2015). This model extended earlier versions (Jørgensen & Fiksen, 2010; Jørgensen & Holt, 2013) by including temperature-dependent physiology and a key physiological constraint through aerobic scope. This model thus has four important trade-offs: (1) foraging more increases predation risk, which is a key trade-off in behavioural ecology and reviewed in Enberg *et al*. (2012); (2) reproducing more increases predation risk, where underlying mechanisms could relate to how developing gonads and sexual display traits, finding mates and mating itself may all be more efficient if the potential for predation is overlooked or downplayed; (3) the ability to escape from predators is compromised when the sum of all metabolic processes approaches the aerobic scope (Priede, 1985; Billerbeck *et al*., 2001; Lankford *et al*., 2001; Claireaux & Chabot, 2016); (4) predation mortality is size dependent, which is well described in marine ecology (Peterson & Wroblewski, 1984; McGurk, 1986; Gislason *et al*., 2010).

In particular, aerobic scope (trade-off 3) has been identified as a key constraint for physiological performance (Eliason *et al*., 2011; Eliason & Farrell, 2016) and is central to the theory for climate adaptations in fishes (Pörtner & Knust, 2007; Pörtner, 2010). A theory for almost an identical trade-off, but related to foraging risk instead of aerobic scope, was also developed in terrestrial ecology (Porter *et al*., 1973, 2000), with predictive ability for species distributions (Agosta *et al*., 2013).

## A different route: merging bioenergetics and community ecology

The empirical and modelling studies described above aim for ever greater detail in describing how the different components of physiology, behaviour and life-history traits fit together, understood through trade-offs that determine the balance between the costs of different functions and their benefits. Typically, however, these are not the best models for addressing strong ecosystem feedback loops. Models help researchers think because they simplify, but as a consequence models cannot resolve all components of natural complexity at the same time. A different route is to describe properties of the ecosystem, with focus on consequences of physiology and its scaling for the structure and productivity of fish communities. Recently, there has been an increasing focus on how the threat of predation can alter several ecological functions through cascades (Schmitz, 2010). In fishes, this is best known from Trinidadian guppies *Poecilia reticulata* Peters 1859. In the earliest experiments, the focus was on how predators affected colouration and sexual selection (Endler, 1980), then life-history traits (Reznick *et al*., 1990) and more recently how predators alter demography, with consequences for competition, the productivity of the resource base and, in turn, life-history traits (Bassar *et al*., 2012).

A marine counterpart to the MTE are size-spectrum models of entire ecosystems, with broad and general assumptions and sweeping conclusions, but with much more emphasis on the structuring processes of predation and growth, and with maintaining consistency in terms of reproduction and fitness (Andersen & Beyer, 2006). Physiologically structured population models detail individual foraging and energetics (de Roos, 1996; Persson *et al*., 1998), much like DEB, but focus more on population dynamics and competitive interactions (Persson *et al*., 2007; Van Leeuwen *et al*., 2008). Yet, other options are ecosystem models that trace the flow of energy and nutrients across entire communities of species, sometimes divided into functional groups (*e*.*g*. Ecopath with Ecosim, Christensen & Walters, 2004 ; Atlantis, Fulton *et al*., 2011). These models put less emphasis on individual traits and physiological mechanisms, but harness instead the wealth of marine data from monitoring time series with the consequence that the models are parameter-rich. Often, they are coupled to physical ocean models for strong links to environmental drivers.

## Conclusions

Great advances were made in behavioural ecology when the perspective of ethology was extended to include survival trade-offs. This allowed two types of consequences of behaviours to be integrated that, until that point, had been understood in isolation: for bioenergetics and for survival. Making this way of thinking pervasive also in physiology may pave the way for a broader integration of physiology, behaviour and life-history theory, and a more holistic approach to understanding why organisms, due to natural selection, have ended up with the traits and properties they have. For all of the trade-offs discussed in this paper, the empirical basis could have been much stronger, and often models need to assume parameter values based on the expected functional shape of a trade-off or other more or less qualified guesstimates. Obviously, the difficulties of observing mortality (Gislason *et al*., 2010) puts aquatic biology at a disadvantage compared with terrestrial disciplines; predation often happens at depth or in darkness, and predation itself or its traces are typically impossible to observe and difficult to estimate. Moreover, the most significant effects of a predator may be non-consumptive, driving changes in activity or behaviour that have other costs than immediate death from being eaten (Schmitz, 2010). Still, ingenious empiricists are able to quantify almost anything with high precision, in the laboratory or in the field, and the challenge is hereby put forward to observationalists of all types, to focus more on trade-offs with predation. With new methods for measurement of field metabolic rate in fishes (Metcalfe, J. D. *et al*., 2016), there is hope for greater integration of bioenergetics, behaviour and functional ecology, interpreted in light of fitness.

Turning to modelling may enforce a stronger focus on context and, faced with this, a practitioner might need to reconsider how their own discipline relates to the rest of biology (Cooke *et al*., 2014). To be successful at interdisciplinary research, a shift has to be made beyond the comfort zone, and a new thing or two might need to be learnt. It is not uncommon that this makes a stronger scientist, who can interact more broadly across disciplines, which is a really good reason for engaging in applied or cross-disciplinary science from time to time.

The authors are grateful to S. Auer, D. Chabot and D. M. McKenzie for critical and constructive comments that improved the paper. C.J. acknowledges financial support from EU for participation in COST Action ‘FA-1004 Conservation Physiology of Marine Fishes’ during which ideas in this paper were developed. K.E. acknowledges financial support from the Research Council of Norway.
